# Additive Complex Ayurvedic Treatment in Patients with Fibromyalgia Syndrome Compared to Conventional Standard Care Alone: A Nonrandomized Controlled Clinical Pilot Study (KAFA Trial)

**DOI:** 10.1155/2013/751403

**Published:** 2013-09-01

**Authors:** Christian S. Kessler, Thomas Ostermann, Larissa Meier, Elmar Stapelfeldt, Silvia Schütte, Joachim Duda, Andreas Michalsen

**Affiliations:** ^1^Immanuel Hospital Berlin, Department of Internal and Complementary Medicine, 14109 Berlin, Germany; ^2^Immanuel Hospital Berlin and Institute of Social Medicine, Epidemiology and Health Economics, Charité University Medical Center Berlin, Königstraße 63, 14109 Berlin, Germany; ^3^Witten-Herdecke University, Medical Faculty, 58448 Witten, Germany; ^4^Charité University Medical Center, Institute of Social Medicine, Epidemiology and Health Economics, 10117 Berlin, Germany

## Abstract

*Background*. Fibromyalgia (FMS) is a challenging condition for health care systems worldwide. Only limited trial data is available for FMS for outcomes of complex treatment interventions of complementary and integrative (CIM) approaches. *Methods*. We conducted a controlled, nonrandomized feasibility study that compared outcomes in 21 patients treated with Ayurveda with those of 11 patients treated with a conventional approach at the end of a two-week inpatient hospital stay. Primary outcome was the impact of fibromyalgia on patients as assessed by the FIQ. Secondary outcomes included scores of pain intensity, pain perception, depression, anxiety, and quality of sleep. Follow-up assessments were done after 6 months. *Results*. At 2 weeks, there were comparable and significant improvements in the FIQ and for most of secondary outcomes in both groups with no significant in-between-group differences. The beneficial effects for both treatment groups were partly maintained for the main outcome and a number of secondary outcomes at the 6-month followup, again with no significant in-between-group differences. *Discussion*. The findings of this feasibility study suggest that Ayurvedic therapy is noninferior to conventional treatment in patients with severe FMS. Since Ayurveda was only used as add-on treatment, RCTs on Ayurveda alone are warranted to increase model validity. This trial is registered with NCT01389336.

## 1. Introduction

Fibromyalgia (FMS) is a complex and challenging condition for health care systems worldwide [1]. FMS patients typically suffer from a range of symptoms that, aside musculoskeletal pain, may also include fatigue, sleeping problems, bowl and bladder disorders, and neurological, psychomental and other symptoms [1–3]. On a global level the prevalence of FMS ranges from 0.7% to 3.3% in adult populations with an increase in recent years and a continuous trend towards female patients [4–6].

Although cause, etiology, and pathodynamics of FMS are still unknown, several hypotheses have been developed including “central sensitization.” This theory proposes that FMS patients have a lower threshold for pain due to increased reactivity of pain-sensitive nerve cells in the spinal cord or brain [1, 7]. Genetic abnormalities with an impact on inflammatory and stress pathways for comorbidities have also been discussed [8]. Research has also demonstrated an abnormal pain processing and lowered mechanical and thermal pain threshold by functional magnetic resonance imaging (fMRI) [9] in FMS patients as well as dysfunction of descending pain modulatory systems, for example, in the rostral anterior cingulate cortex (rACC) [10], and distinct neurotransmitter activities in the cerebrospinal fluid (CF) [11]. Further discovered dysfunctions of the neuroendocrine axis may also serve as a hypothesis for concomitant symptoms of FMS that are predominant in most of the FMS patients [12]. A close association with psychosocial stress seems to be likely [12, 13]. Evidence suggests that the pain in FMS results primarily from pain processing pathways functioning abnormally. In simple terms it can be described as the amount of neurons being set too high, and these hyperexcitability of pain processing pathways and underactivity of inhibitory pain pathways in the brain result in the affected individual experiencing pain [14]. 

Recent FMS guidelines therefore recommend a multimodal, multidisciplinary therapeutic approach involving medication, exercise, patient education, and behavioral and psychosomatic therapies [3, 15]. Due to frequent unsatisfying results of conventional medicine (CoM) treatment a substantial proportion of patients use complementary and integrative (CAM/CIM) approaches such as mind-body medicine, nutritional supplements, phytotherapy, acupuncture, massage, various nutritional therapies, and traditional and whole medical systems (WMS) like traditional Chinese medicine (TCM) or Ayurveda [16].

The traditional Indian WMS Ayurveda has been experiencing a resurgence in popularity in its native countries (in particular in India and Sri Lanka) and abroad (in particular in Europe and North America) as it has become more accessible and more in demand [17]. Ayurveda is the most prominent traditional medical (TM) system in South Asia [18]. In India WMS Ayurveda is recognized by the state, legally put on par with CoM, and used in an area with more than 1.4 billion people as a broad system of medicine and is fully recognized by the World Health Organization (WHO) as a medical science [19–21]. The importance of Ayurveda is reflected by the fact that more than 400,000 Ayurvedic physicians are registered in India alone [22]. Over 250 universities and colleges are recognized by the Indian Government where Ayurvedic medicine is systematically taught, practiced, and promoted by the state [23]. Moreover, Ayurvedic clinical research has begun to find its way into mainstream medical journals recently [18]. The past decade has seen some important clinical trials that point towards future directions [17, 24–26]. Nevertheless, the evaluation of WMS Ayurveda with the tools from evidence-based medicine (EbM) is still in a pioneering stage in spite of a growing number of clinical trials and the launch of digital science databases for research on Ayurveda [27].

In Germany, several academic hospital departments for naturopathic and integrative medicine have accumulated clinical experience in inpatient treatments of FMS. Within the treatment concepts of integrative approaches, Ayurveda is one of the fastest growing CAM systems in this context [18] and is being used in several university associated inpatient hospital setups in Germany, for example, in Berlin, Essen, and Hattingen [28].

Clinical experience and preliminary evidence from uncontrolled prospective studies suggest that a CIM approach focusing on Ayurveda may help to decrease symptoms of FMS [29–32]. However, it would be useful to know how such an approach would act, if compared with CoM FMS treatment, as established in specialized hospital units of rheumatology or pain medicine. We thus conducted a controlled nonrandomized feasibility study comparing a CIM treatment strategy focusing on Ayurveda with a conventional rheumatologic treatment strategy for FMS in order to evaluate and analyze potential strength, weaknesses, opportunities, and safety issues of the CIM intervention in the process of decision making and methodological planning for further trials in this field.

## 2. Materials and Methods

### 2.1. Study Design and Participants

The study was conducted as a prospective, controlled nonrandomized feasibility study. The study protocol was reviewed and approved by the Ethics Committee of the Charité University Medical Center, Berlin, Germany, and all patients gave informed consent for their study participation. trial registration was done at clinical Trials (registration no. NCT01389336, acronym: KAFA trial) [33]. Collection of data was performed by trained study personnel. 

All trial participants were inpatients during a 16-month period from two different departments of the Immanuel Hospital Berlin, a hospital specialized in the treatment of rheumatic and chronic pain diseases:

- patients of the Department of Integrative and Complementary Medicine
- patients of the Department of Internal Medicine and Rheumatology.

The study sample consisted of consecutively admitted inpatients with primary diagnosis of FMS as reason for inpatient hospital admission. Patients regularly stayed 14 ± 2 days in hospital for multidisciplinary treatment, which is in full accordance with the German DRG system [34].

Inclusion criteria were a manifested and prediagnosed FMS according to the ACR 2010 criteria [35] and the current German guidelines, as diagnosed by a rheumatologist, pain specialist, or internist and age between 18 and 65 years. Exclusion criteria were recent change of FMS pharmacotherapy ≤ 6 weeks ago, acute psychotic conditions, current intake of opioid analgesics (intake of an other medication with known efficacy for FMS, e.g., SNRIs or anticonvulsants, was not an exclusion criterion), currently undergoing hyperthermia treatment, existing coagulation disorders, intake of coagulation-inhibiting medication other than acetylsalicylic acid and clopidogrel, being in the process of applying for pension or disability benefits, pregnancy or breastfeeding, obesity ≥ grade II, severe comorbidity (e.g., heart failure NYHA IV, cancer, or AIDS), simultaneous participation in another clinical trial, and participation in another clinical trial ≤ 6 month ago.

### 2.2. Interventions

The CoM treatment consisted of a complex multidisciplinary treatment approach with the following elements: group physiotherapy, hydrotherapy, thermal therapy, psychosomatic therapy, aerobic exercise, pool exercise, cognitive behavioral therapy education, occupational therapy, and specific pain therapy. The CIM treatment used the same treatment elements as described previously. In addition, patients received complex tailored Ayurvedic treatment according to the Ayurveda diagnosis which included manual treatments, massages, dietary advice, Ayurvedic lifestyle, and yoga posture advice and instructions for daily self-applied massage. Patients of both groups received the same total time amount of treatments with a total of 1600 to 2200 treatment minutes in the 14 ± 2 days of the hospital-stay period, according to the German DRG system. Thereby both groups received the same spectrum of multimodal conventional treatment modalities; however, in the Ayurveda group, some amount of time for CoM treatments was cut down and replaced by Ayurvedic treatments.

### 2.3. FMS from the Ayurvedic Perspective

**Figure 1:**
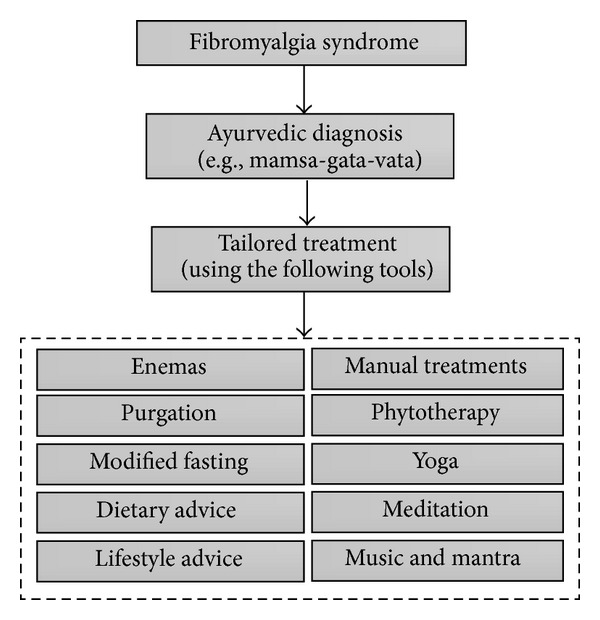
Classic Ayurvedic treatment of fibromyalgia (simplified depiction).

Ayurveda has differentiated hermeneutic concepts that form the basis for diagnosis and therapy of FMS. Here, central theoretical foundations are the dosha and guna principles, two core concepts of Ayurvedic nosology. Foremost, the three doshas, vata, pitta, and kapha, are considered to be superior regulating principles of physiological and pathophysiological processes in the organism. Additionally, the three gunas, sattva, rajas, and tamas, regulate the psychomental realm [34]. These classical Ayurvedic approaches describe postulated dynamic flow systems with constant interactions between the aforementioned principles. For a further understanding of Ayurvedic medicine, it is essential not to interpret these principles as trivial “energies” in a physical and/or metaphysical sense but rather as explanatory models that are used metaphorically in order to depict the complexity of the body-mind milieu and to categorize phenomena in general [33]. As for FMS (Ayurvedic diagnostic approximation: mamsa-gata-vata), vata and tamas especially are relevant to the interpretation, diagnosis, and treatment of the disease entity, and, from the Ayurvedic viewpoint, the correction of “*milieu interieur*” changes [17]. For a general outline of Ayurvedic FMS strategies, see Figure 1.

### 2.4. Measurements

All measures were assessed by trained study nurses at three study visits: at baseline, after 2 weeks (at dismissal from hospital), and at a 6-month followup. The primary outcome measure was the change in the Fibromyalgia Impact Questionnaire (FIQ) score from baseline to the end of the inhospital intervention. The FIQ is a validated, multidimensional measure to assess the severity of FMS as rated by patients. The total score ranges from 0 to 100, with higher scores indicating more severe symptoms [36]. In this study, the validated German version was used [37].

Global pain status was assessed additionally by asking the patients for the global severity of the disease-related pain by means of a self-rating 10-point Numeric Rating Scale (NRS) with a value of 10 indicating maximum pain and 0 indicating no pain. 

Prespecified other secondary outcomes included (1) a 100 mm visual analogue scale for self-rated global quality of sleep; (2) the German version of the Spielberger State-Trait Anxiety Inventory (STAI), which consists of 20 items related to state anxiety and 20 items related to trait anxiety [38]; (3) the German version of the Hospital Anxiety and Depression Scale (HADS) [39], a validated standard measure for anxiety and depression which uses a 14-item scale with seven of the items being related to anxiety and seven items being related to depression [40]; and the German version of the Pain Perception Scale (SES), which assesses sensory pain perception in chronic pain patients [41].

Subjects height and body weight were measured following a standardized protocol, while patients wore light clothing and no shoes after an overnight fast. BMI was calculated as weight (kg)/height^2^ (m). Anthropometrical and clinical data were collected by trained study personnel. Seated blood pressure was measured after 5 min rest with a calibrated sphygmomanometer at the nondominant arm by trained nurses.

### 2.5. Statistical Analysis

As the study was designed as a nonrandomized feasibility study, no sample size calculation was conducted. However, we intended to include 40 patients and assumed a dropout rate of 15%. Baseline differences were calculated using two tailed *t*-tests. All outcome criteria were analyzed per protocol. Treatment effects were estimated within these models and reported as group differences including their respective 95% confidence intervals (CI) and *P* values. All reported *P* values were based on two-sided *t*-tests, and a *P* value < 0.05 was considered significant. All statistical computations were performed with SPSS statistical software version 21.

## 3. Results

### 3.1. Baseline

During the 16-month study recruitment period, we screened 60 patients with manifested FMS who were admitted to one of the two hospital departments. Of these, 32 fulfilled the eligibility criteria and volunteered to participate in our study: 11 in the Department of Rheumatology and 21 in the Department of Integrative and Complementary Medicine. Data assessments were done on site at study visits 1 (baseline) and 2 (week 2) and by post after 6 months by the Department of Integrative and Complementary Medicine. Notably, the comparatively small number of patients in the CoM group is explained by the fact that a large number of screened patients in the rheumatology department could not be included into the trial, in particular due to obesity or applications for pension or disability benefits. Baseline characteristics of the study population revealed a middle-aged and only female study population. Patients of the CoM group showed a higher FIQ score, the primary outcome, and had slightly higher pain scores compared to patients of the CIM group (Table 1).

### 3.2. Primary Outcome

The FIQ score decreased significantly both in the CIM group and in the CoM group after 2 weeks, with between-group differences being not significantly different (Table 2). At 6th month, the FIQ score increased again in both groups, while differences to baseline remained significant within groups. Again, no significant differences between the groups could be detected (Table 2).

### 3.3. Secondary Outcomes

At 2 weeks, the integrative medicine group had greater mean improvements in all secondary outcomes except the STAI state score (Table 2). The HADS depression score improved significantly better in the CIM group compared to the CoM group. All other 2 week differences between the groups were not statistically significant.

At 6 months, scores showed a trend towards beneficial midterm outcomes for both groups without significant differences between the two groups. In particular, the STAI and HADS scores suggested a better midterm treatment effect in the CIM group. Most of the outcomes deteriorated again compared to the 2-week results resulting globally in only mild treatment effects compared to baseline levels (Table 2).

### 3.4. Safety

There were no serious adverse events in both groups. About 25% in each group reported some minor side effects. Within the CIM group, the first treatment days were frequently accompanied by tiredness, mood changes, and muscle pain. Patients in the CoM group reported frequently muscle pain and tiredness, most likely due to exercise and physical therapies. Treatment seemed to be safe in both groups.

## 4. Discussion

In this controlled nonrandomized feasibility trial, we compared the effectiveness of two time- and attention-balanced inpatient complex treatment strategies for FMS: an integrative medicine approach focusing on Ayurveda medicine (CIM) versus conventional rheumatologic therapy (CoM). While patients in the CoM group were more diseased at baseline, adjusted data analysis showed a slightly more beneficial effect of the CIM approach after 2 weeks for all clinical outcomes, which were, however, not statistically significant and not clinically relevant to almost all outcomes except for the HADS depression scale. At month 6, treatment effects in both groups were reduced with still significant within-group differences for most of the outcomes including the primary outcome FIQ. However, no statistically significant differences between the groups could be observed here. 

Looking at the overall still unsatisfying treatment options for FMS, new therapeutic approaches are needed. At the same time, the majority of FMS patients frequently use CAM methods alongside conventional treatment. In Germany, CAM and WMS like Ayurveda as part of CAM are very popular. Among those, Ayurveda is one of the fastest growing CAM systems [17, 18]. First and small clinical trials have hinted at a possible effectiveness of Ayurveda in the treatment of FMS, yet the available data is still too weak to draw definite conclusions as these trials were uncontrolled [29–32] and included meditation as part of the Ayurveda-labeled treatment [29, 30]. Several Ayurveda trials have been done on other disease entities from the field of rheumatology, for example, on rheumatoid arthritis and osteoarthritis; here the quality of data seems to be clearly better (e.g., [17, 24, 25]). However, to date, no study has investigated an integrative multimodal treatment program for FMS that focuses on Ayurveda. 

Several limitations relate to our study. First, we used a nonrandomized open label study design as it is not currently possible to randomize patients to hospital departments when costs are covered by health insurance companies under usual care. Nonrandomized studies may introduce a bias by patient selection and different prognostic and response factors between the groups. In this context, the placebo effect is a source of potential bias, in particular in open studies with no attempt at blinding, as in this study; the fact that patients with a preference for CAM could choose this form of treatment compounds this issue and is another limitation of this trial. In fact, baseline values found patients of the CoM department to be overall more diseased than the CIM population. However, baseline differences were statistically nonsignificant, and all our data analysis included baseline values as covariates. Of note, physicians can refer patients to both hospital departments only if they are documented as nonresponders to intensive outpatient treatment. The selection of the department (rheumatology or integrative medicine) is mainly influenced by patients' preference. Here a specific selection bias may be introduced as patients interested in integrative medicine are possibly more likely to search for comprehensive treatments in less severe disease states. Second, our study population was of limited size, in particular in the CoM group, where a lot of screened patients unfortunately had to be excluded. Smaller study populations hold the risk of overestimation of effects on the one side and nondetection of moderate treatment effects on the other side, intensified by different group sizes. A third limitation is the short observation period of 6 months. Further studies should include long-term observation periods with followups, for example, after 12 months and 24 months. Another limitation of this trial is an obvious gender bias, since only female participants were included in this trial.

Another point of discussion is the fact that the patients in the CoM group exhibited a remarkably low improvement on pain (NRS) at least at their third visit. This may be mainly due to the selection of the patients that are only referred to hospital-based inpatient treatment if they are documented as nonresponders to extensive outpatient treatments. In addition, these chronic pain patients often show dissociations between subjective pain ratings and function. 

In view of our documented effects and safety of the CIM approach with a focus on Ayurveda, further research on its effectiveness in FMS is warranted. Moreover, studies on Ayurveda as a WMS alone compared to standard treatment of FMS are warranted. In both cases, such trials should have a larger sample sizes and allocate patients randomly. Since Ayurveda was only used as an add-on treatment within a multimodal CIM package treatment in this trial, the information value of this feasibility trial data for Ayurveda as a WMS is limited, in particular for the Ayurvedic phytotherapy which was not used in this trial. RCTs on WMS Ayurveda care alone in the intervention group are warranted to increase model validity in this case and as a general rule for future clinical research on Ayurvedic medicine.

Strengths of our study relate to the fact that both departments are situated in the same hospital and that, besides fasting and mind-body medicine, all other treatments were comparable and applied by the same personnel. Thus setting effects, attention effects, and other nonspecific factors that may otherwise introduce bias in comparative studies were minimized.

In conclusion, our preliminary findings from this feasibility study indicate that a multimodal CIM treatment with a focus on Ayurveda might be noninferior to the multimodal rheumatology CoM approach in the short- and midterm inpatients with severe FMS. 

## Figures and Tables

**Table 1:** Baseline characteristics.

Characteristics	Integrative medicine group	Rheumatology group	*P* value
Male/female, no.	0/21	0/11	n.a.
Age, years	54.4 ± 9.3	47.8 ± 13.6	0.11
Body mass index, kg/m^2^	26.8 ± 4.5	28.0 ± 4.4	0.46
SBP, mmHg	128 ± 14	129 ± 10	0.77
DBP, mmHg	75.3 ± 9.3	78.1 ± 8.0	0.46
FIQ score	46.9 ± 12.6	51.0 ± 12.2	0.38
Pain score (NRS)	6.0 ± 1.9	6.7 ± 2.5	0.34
Quality of sleep	4.0 ± 1.9	3.5 ± 3.1	0.59
STAI state score	52.4 ± 6.0	50.7 ± 11.2	0.65
STAI trait score	45.9 ± 11.5	46.8 ± 11.4	0.83
HADS anxiety	8.8 ± 3.6	9.0 ± 3.8	0.89
HADS depression	7.4 ± 3.4	7.4 ± 3.6	0.96
SES sensory pain perception	20.9 ± 5.8	20.7 ± 6.0	0.95
SES affective pain perception	31.7 ± 8.4	34.6 ± 9.2	0.38

Values are mean ± SD if not indicated otherwise. SBP: systolic blood pressure; DBP: diastolic blood pressure.

STAI: state and trait anxiety questionnaire, FIQ: fibromyalgia impact questionnaire; HADS: hospital anxiety and depression scale; NRS: numeric rating scale 0–10; SES: pain perception scale.

**Table 2:** Outcomes in both groups at baseline, week 2 and month 6 with group differences as indicators of change.

	Integrative medicine group			Conv. rheumatology group			Mean difference		Mean diff. (95% CI)	
	Baseline (*n* = 21)	Visit 2 (*n* = 20)	Visit 3 (*n* = 18)	Baseline (*n* = 11)	Visit 2 (*n* = 11)	Visit 3 (*n* = 7)	∇ 1-2 (95% CI)	*P* value	∇ 1–3 (95% CI)	*P* value
FIQ score	46.9 ± 12.6	32.8 ± 14.0	36.5 ± 17.1	51.0 ± 12.2	38.1 ± 8.7	40.7 ± 14.6	1.30 (−6.00; 8.59)	0.72	2.97 (−6.26; 12.20)	0.51
Pain score (NRS)	6.0 ± 1.9	4.2 ± 2.5	4.2 ± 2.8	6.7 ± 2.5	5.7 ± 2.1	6.3 ± 2.3	0.89 (−0.87; 2.65)	0.31	2.08 (−0.52; 4.69)	0.11
Quality of sleep	4.0 ± 1.9	4.7 ± 2.7	5.1 ± 2.5	3.5 ± 3.1	4.5 ± 2.7	5.3 ± 3.9	0.09 (−2.52; 2.70)	0.94	0.17 (−2.99; 3.33)	0.91
HADS anxiety	8.8 ± 3.6	5.7 ± 3.7	8.1 ± 3.4	9.0 ± 3.8	8.1 ± 4.3	9.3 ± 4.8	2.09 (−0.66; 4.84)	0.13	1.48 (−2.05; 5.00)	0.40
HADS depression	7.4 ± 3.4	4.3 ± 2.4	7.1 ± 2.5	7.4 ± 3.6	7.3 ± 4.0	9.7 ± 5.0	2.86 (0.67; 5.05)	0.01	2.43 (−0.37; 5.22)	0.09
SES sensory pain perception	20.9 ± 5.8	16.6 ± 4.0	19.8 ± 6.8	20.7 ± 6.0	21.1 ± 7.2	19.5 ± 5.2	0.61 (−4.73; 5.95)	0.82	2.72 (−3.97; 9.40)	0.41
SES affective pain perception	31.7 ± 8.4	26.4 ± 9.6	28.9 ± 10.6	34.6 ± 9.2	30.0 ± 7.9	36.0 ± 11.1	4.61 (−0.15; 9.38)	0.06	0.87 (−5.06; 6.80)	0.76
STAI state score	52.4 ± 6.0	44.1 ± 14.2	49.6 ± 8.6	50.7 ± 11.2	42.1 ± 7.0	53.9 ± 13.0	−0.44 (−9.75; 8.87)	0.92	7.97 (−0.19; 16.13)	0.06
STAI trait score	45.9 ± 11.5	39.7 ± 10.4	44.3 ± 11.1	46.8 ± 11.4	45.4 ± 11.2	50.7 ± 13.0	4.40 (−4.14; 12.93)	0.30	6.44 (−7.08; 19.95)	0.33

Values are mean ± SD if not indicated otherwise. SBP: systolic blood pressure; DBP: diastolic blood pressure.

STAI: state and trait anxiety questionnaire, FIQ: fibromyalgia impact questionnaire; NRS: numeric rating scale 0–10; HADS: hospital anxiety and depression scale; ∇ 1-2: difference between groups from baseline to visit 2 at 2 weeks, ∇ 1–3: difference between groups from baseline to visit 3 at 12 weeks. SES: pain perception scale.

*P values for between-group difference of change are adjusted.
